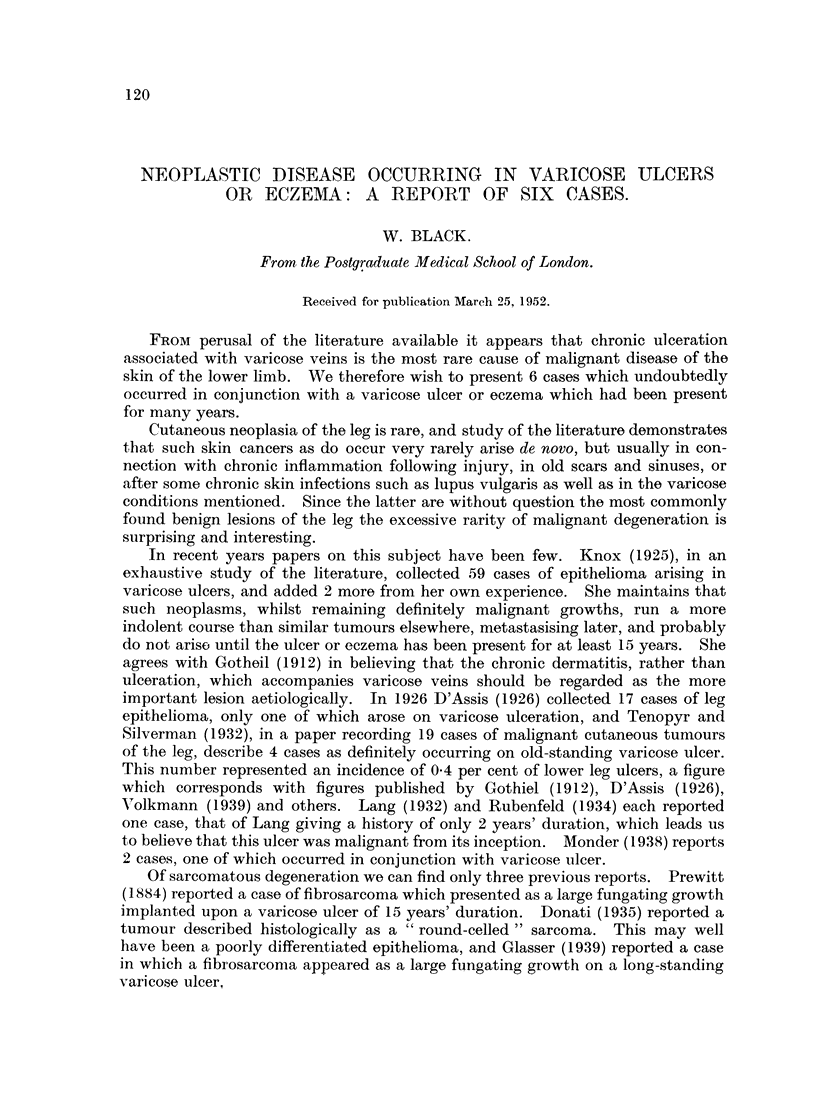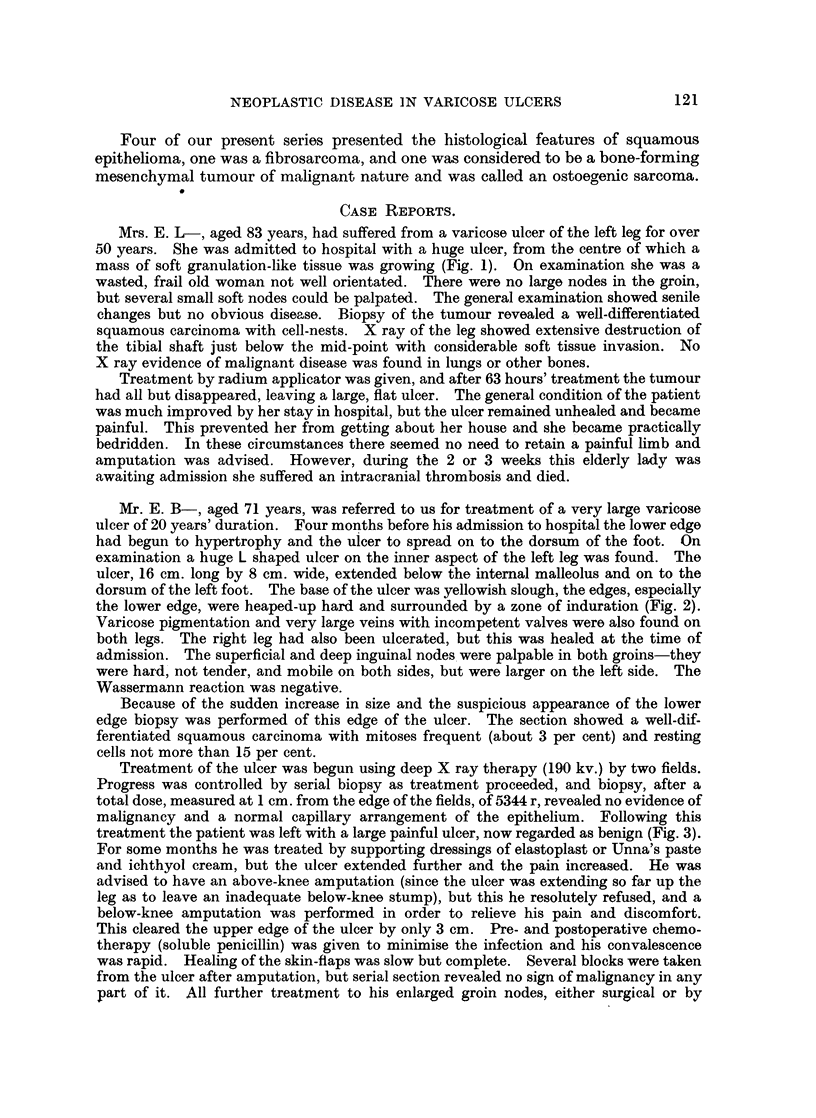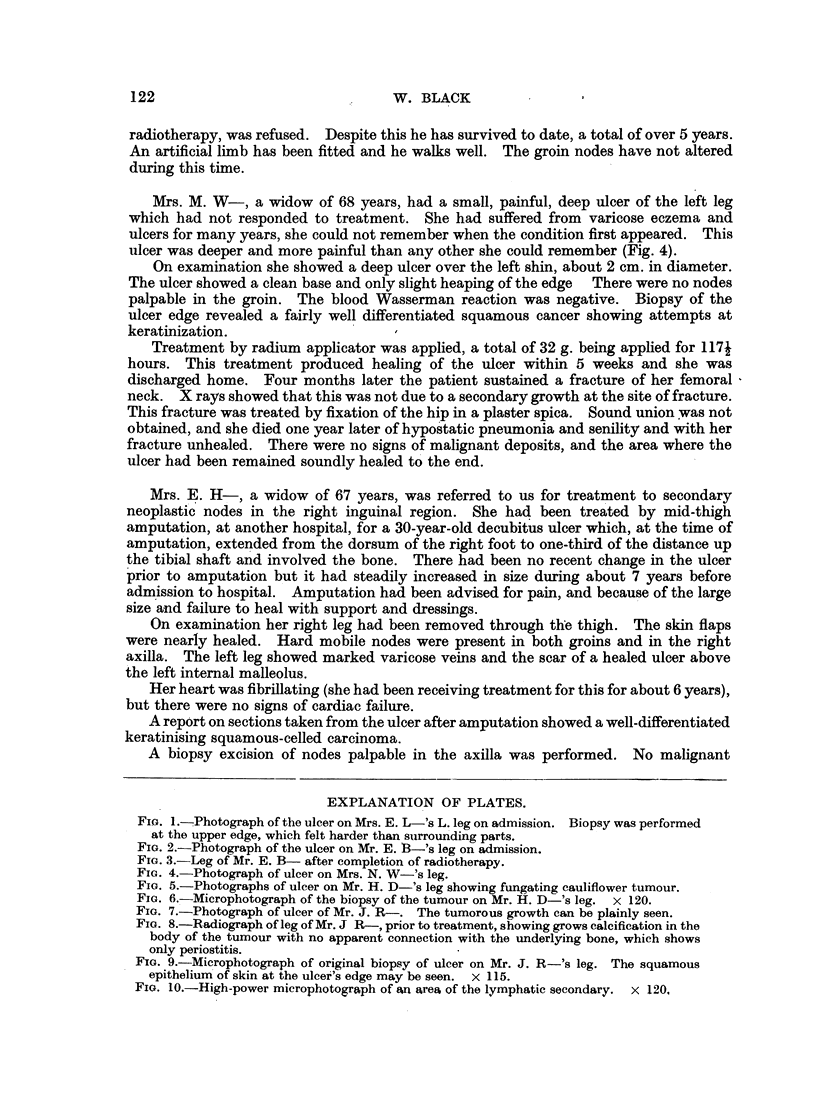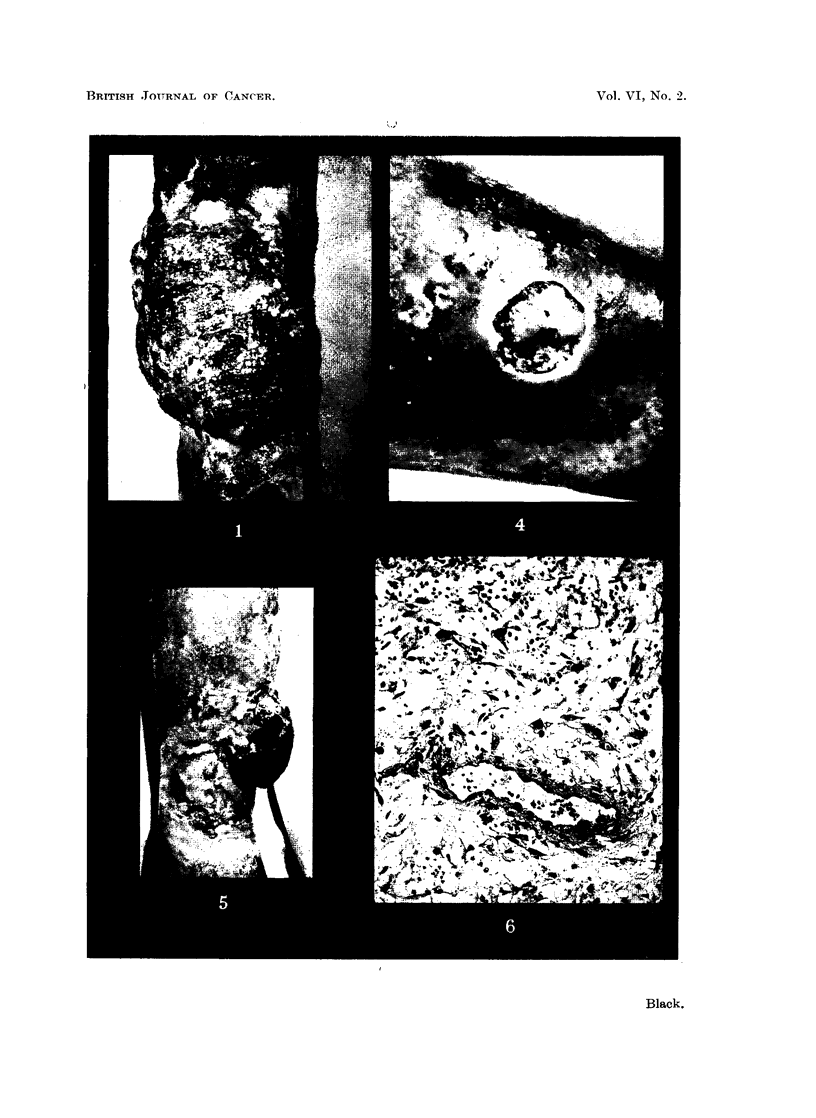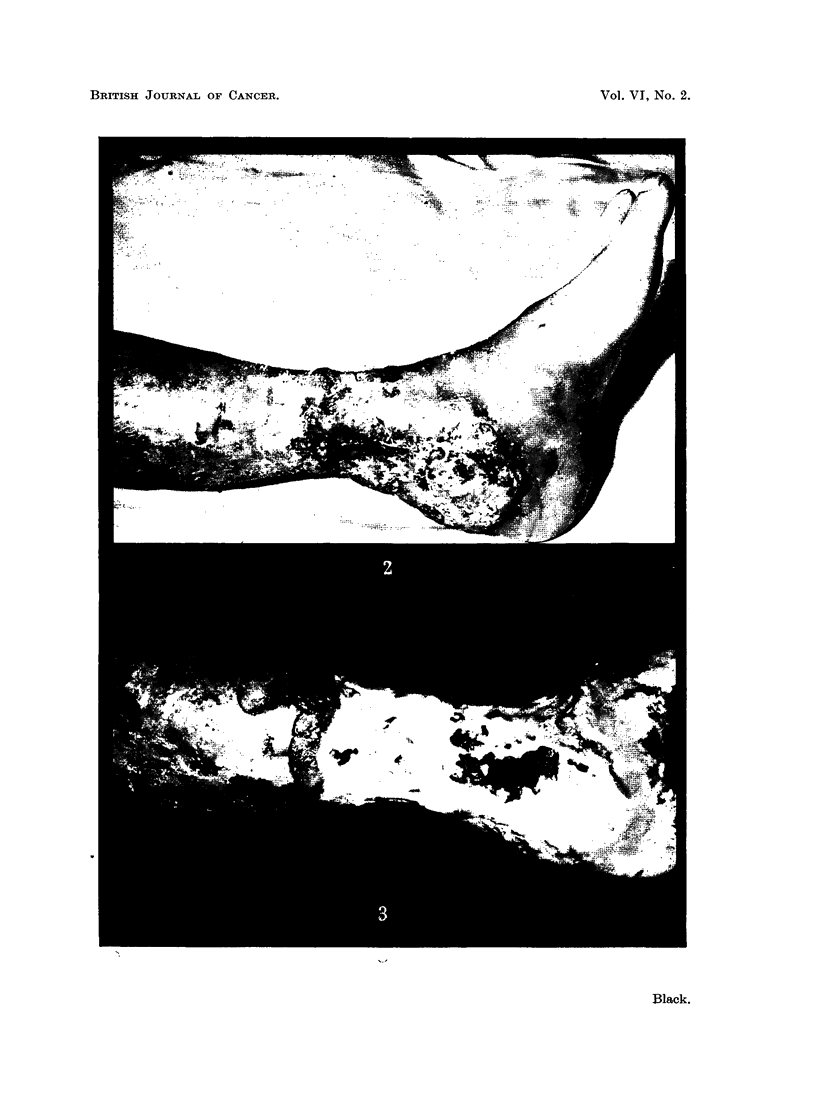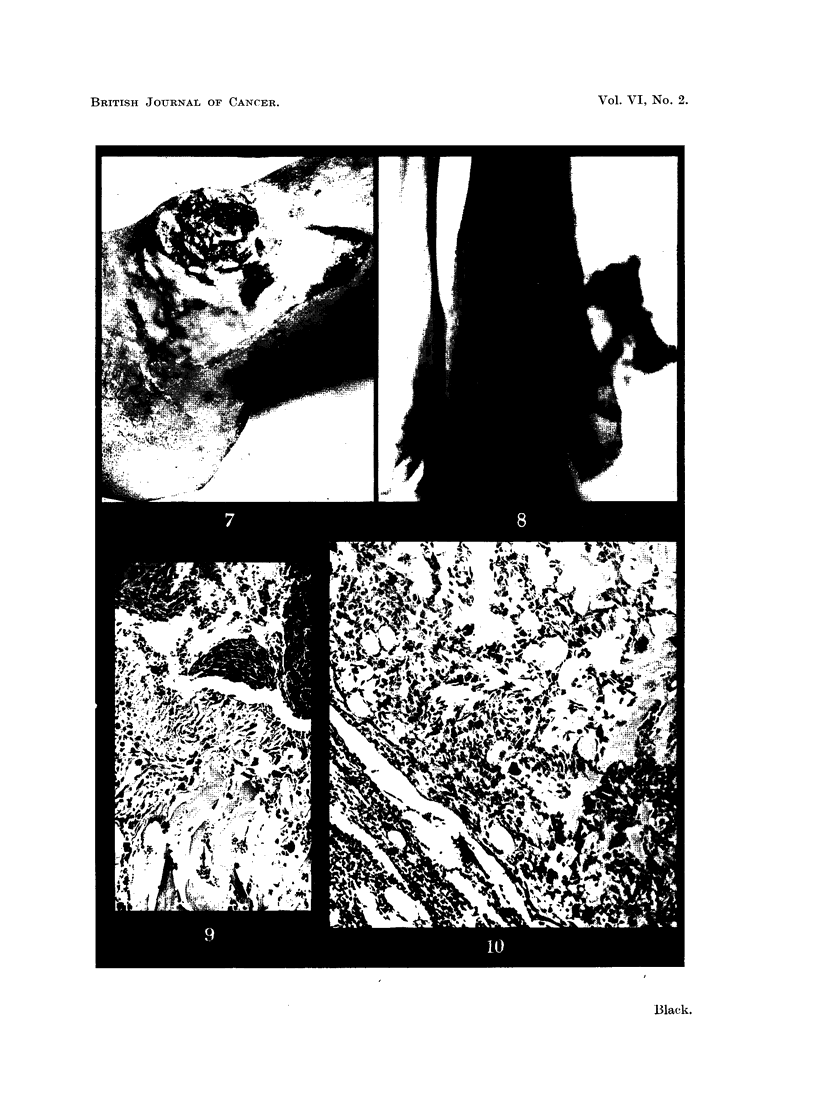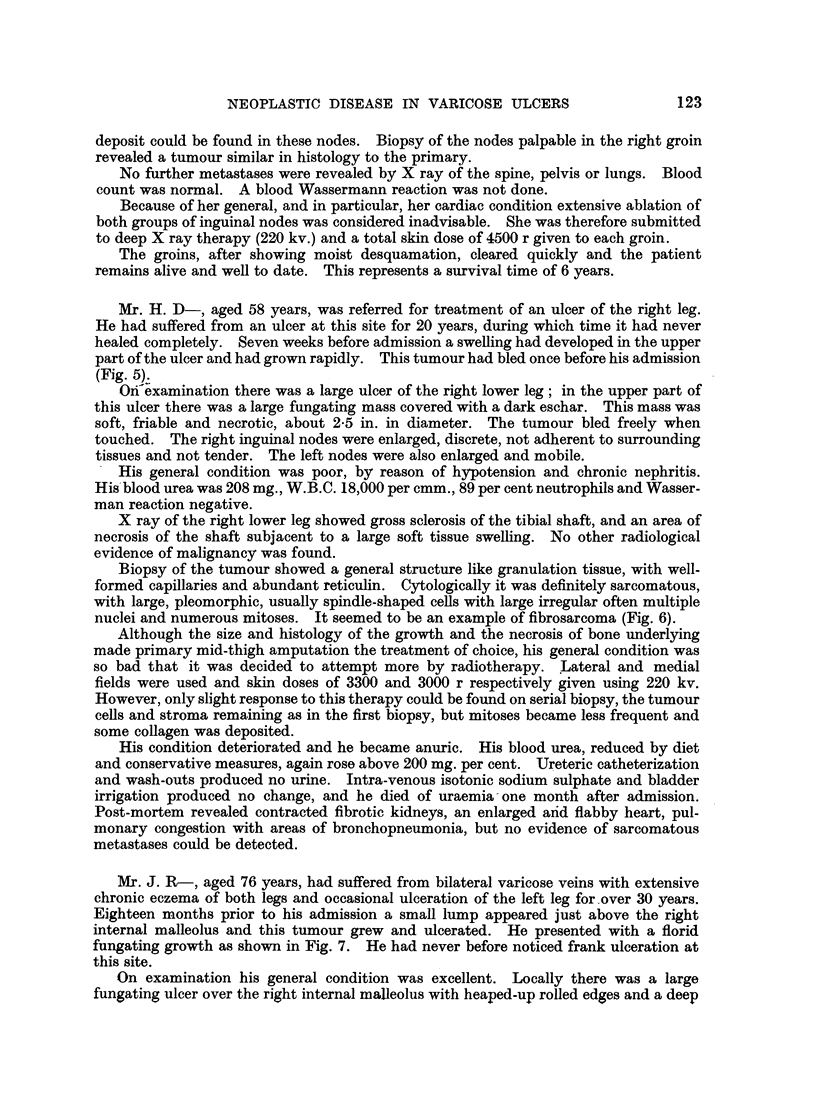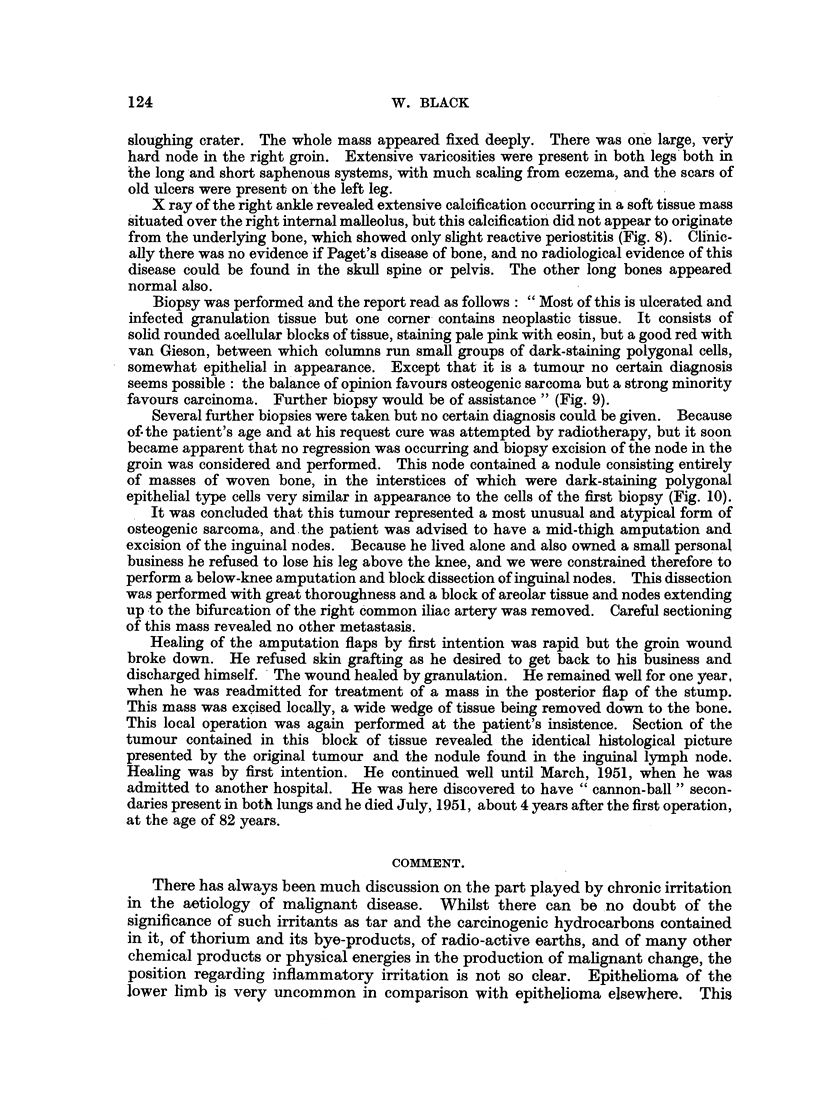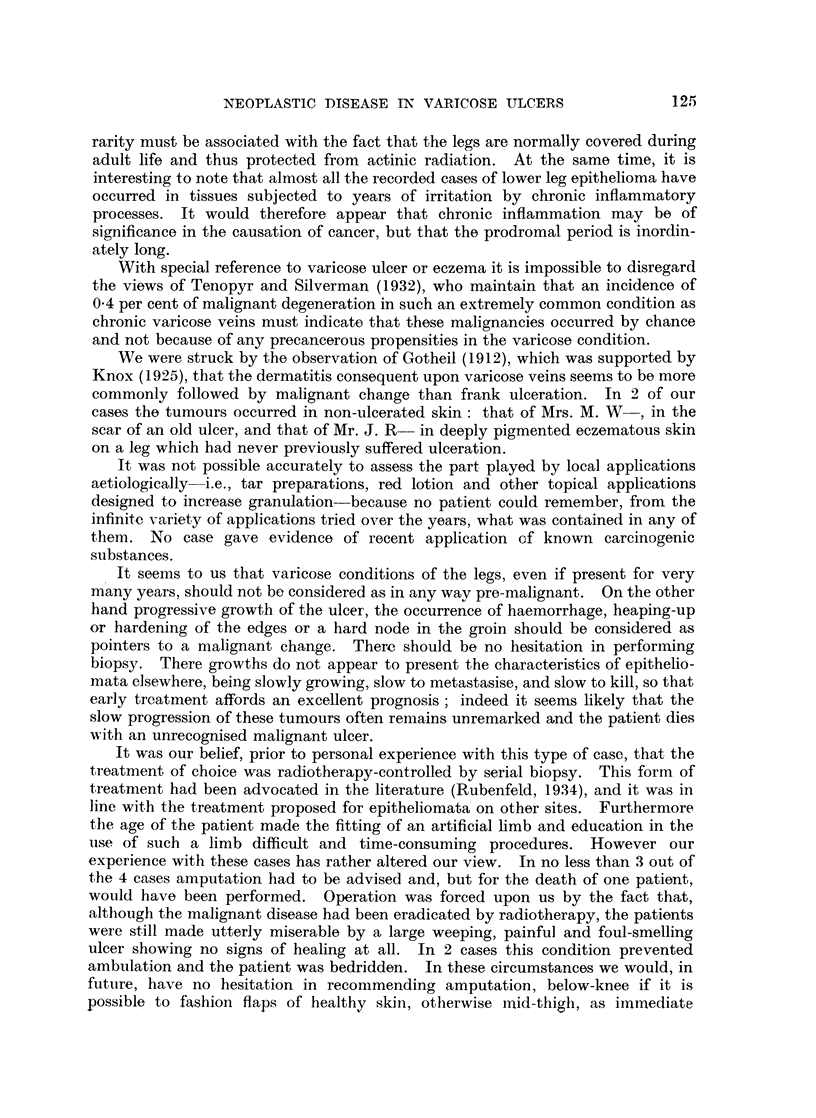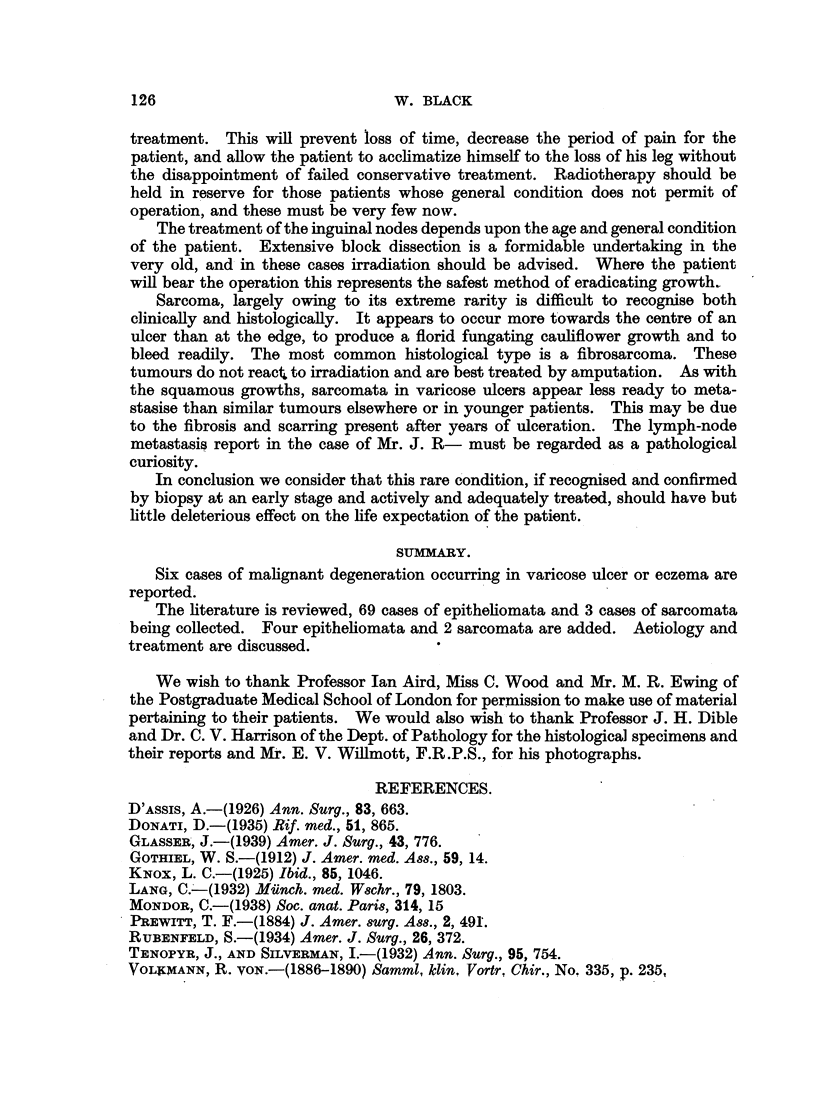# Neoplastic Disease Occurring in Varicose Ulcers or Eczema: A Report of Six Cases

**DOI:** 10.1038/bjc.1952.13

**Published:** 1952-06

**Authors:** W. Black

## Abstract

**Images:**


### NEOPLASTIC DISEASE OCCURRING IN VARICOSE ULCERS OR ECZEMA: A REPORT OF SIX CASES.

W. BLACK.

*From the Postgraduate Medical School of London.*

Received for publication March 25, 1952.

FROM perusal of the literature available it appears that chronic ulceration associated with varicose veins is the most rare cause of malignant disease of the skin of the lower limb. We therefore wish to present 6 cases which undoubtedly occurred in conjunction with a varicose ulcer or eczema which had been present for many years.

Cutaneous neoplasia of the leg is rare, and study of the literature demonstrates that such skin cancers as do occur very rarely arise *de novo,* but usually in connection with chronic inflammation following injury, in old scars and sinuses, or after some chronic skin infections such as lupus vulgaris as well as in the varicose conditions mentioned. Since the latter are without question the most commonly found benign lesions of the leg the excessive rarity of malignant degeneration is surprising and interesting.

In recent years papers on this subject have been few. Knox (1925), in an exhaustive study of the literature, collected 59 cases of epithelioma arising in varicose ulcers, and added 2 more from her own experience. She maintains that such neoplasms, whilst remaining definitely malignant growths, run a more indolent course than similar tumours elsewhere, metastasising later, and probably do not arise until the ulcer or eczema has been present for at least 15 years. She agrees with Gotheil (1912) in believing that the chronic dermatitis, rather than ulceration, which accompanies varicose veins should be regarded as the more important lesion aetiologically. In 1926 D'Assis (1926) collected 17 cases of leg epithelioma, only one of which arose on varicose ulceration, and Tenopyr and Silverman (1932), in a paper recording 19 cases of malignant cutaneous tumours of the leg, describe 4 cases as definitely occurring on old-standing varicose ulcer. This number represented an incidence of 0·4 per cent of lower leg ulcers, a figure which corresponds with figures published by Gothiel (1912), D'Assis (1926), Volkmann (1939) and others. Lang (1932) and Rubenfeld (1934) each reported one case, that of Lang giving a history of only 2 years' duration, which leads us to believe that this ulcer was malignant from its inception. Monder (1938) reports 2 cases, one of which occurred in conjunction with varicose ulcer.

Of sarcomatous degeneration we can find only three previous reports. Prewitt (1884) reported a case of fibrosarcoma which presented as a large fungating growth implanted upon a varicose ulcer of 15 years' duration. Donati (1935) reported a tumour described histologically as a " round-celled " sarcoma. This may well have been a poorly differentiated epithelioma, and Glasser (1939) reported a case in which a fibrosarcoma appeared as a large fungating growth on a long-standing varicose ulcer.

Four of our present series presented the histological features of squamous epithelioma, one was a fibrosarcoma, and one was considered to be a bone-forming mesenchymal tumour of malignant nature and was called an ostoegenic sarcoma.

### Case Reports.

Mrs. E. L—, aged 83 years, had suffered from a varicose ulcer of the left leg for over 50 years. She was admitted to hospital with a huge ulcer, from the centre of which a mass of soft granulation-like tissue was growing (Fig. 1). On examination she was a wasted, frail old woman not well orientated. There were no large nodes in the groin, but several small soft nodes could be palpated. The general examination showed senile changes but no obvious disease. Biopsy of the tumour revealed a well-differentiated squamous carcinoma with cell-nests. X ray of the leg showed extensive destruction of the tibial shaft just below the mid-point with considerable soft tissue invasion. No X ray evidence of malignant disease was found in lungs or other bones.

Treatment by radium applicator was given, and after 63 hours' treatment the tumour had all but disappeared, leaving a large, flat ulcer. The general condition of the patient was much improved by her stay in hospital, but the ulcer remained unhealed and became painful. This prevented her from getting about her house and she became practically bedridden. In these circumstances there seemed no need to retain a painful limb and amputation was advised. However, during the 2 or 3 weeks this elderly lady was awaiting admission she suffered an intracranial thrombosis and died.

Mr. E. B—, aged 71 years, was referred to us for treatment of a very large varicose ulcer of 20 years' duration. Four months before his admission to hospital the lower edge had begun to hypertrophy and the ulcer to spread on to the dorsum of the foot. On examination a huge L shaped ulcer on the inner aspect of the left leg was found. The ulcer, 16 cm. long by 8 cm. wide, extended below the internal malleolus and on to the dorsum of the left foot. The base of the ulcer was yellowish slough, the edges, especially the lower edge, were heaped-up hard and surrounded by a zone of induration (Fig. 2). Varicose pigmentation and very large veins with incompetent valves were also found on both legs. The right leg had also been ulcerated, but this was healed at the time of admission. The superficial and deep inguinal nodes were palpable in both groins—they were hard, not tender, and mobile on both sides, but were larger on the left side. The Wassermann reaction was negative.

Because of the sudden increase in size and the suspicious appearance of the lower edge biopsy was performed of this edge of the ulcer. The section showed a well-differentiated squamous carcinoma with mitoses frequent (about 3 per cent) and resting cells not more than 15 per cent.

Treatment of the ulcer was begun using deep X ray therapy (190 kv.) by two fields. Progress was controlled by serial biopsy as treatment proceeded, and biopsy, after a total dose, measured at 1 cm. from the edge of the fields, of 5344 r, revealed no evidence of malignancy and a normal capillary arrangement of the epithelium. Following this treatment the patient was left with a large painful ulcer, now regarded as benign (Fig. 3). For some months he was treated by supporting dressings of elastoplast or Unna's paste and ichthyol cream, but the ulcer extended further and the pain increased. He was advised to have an above-knee amputation (since the ulcer was extending so far up the leg as to leave an inadequate below-knee stump), but this he resolutely refused, and a below-knee amputation was performed in order to relieve his pain and discomfort. This cleared the upper edge of the ulcer by only 3 cm. Pre- and postoperative chemotherapy (soluble penicillin) was given to minimise the infection and his convalescence was rapid. Healing of the skin-flaps was slow but complete. Several blocks were taken from the ulcer after amputation, but serial section revealed no sign of malignancy in any part of it. All further treatment to his enlarged groin nodes, either surgical or by radiotherapy, was refused. Despite this he has survived to date, a total of over 5 years. An artificial limb has been fitted and he walks well. The groin nodes have not altered during this time.

Mrs. M. W—, a widow of 68 years, had a small, painful, deep ulcer of the left leg which had not responded to treatment. She had suffered from varicose eczema and ulcers for many years, she could not remember when the condition first appeared. This ulcer was deeper and more painful than any other she could remember (Fig. 4).

On examination she showed a deep ulcer over the left shin, about 2 cm. in diameter. The ulcer showed a clean base and only slight heaping of the edge There were no nodes palpable in the groin. The blood Wasserman reaction was negative. Biopsy of the ulcer edge revealed a fairly well differentiated squamous cancer showing attempts at keratinization.

Treatment by radium applicator was applied, a total of 32 g. being applied for 117½ hours. This treatment produced healing of the ulcer within 5 weeks and she was discharged home. Four months later the patient sustained a fracture of her femoral neck. X rays showed that this was not due to a secondary growth at the site of fracture. This fracture was treated by fixation of the hip in a plaster spica. Sound union was not obtained, and she died one year later of hypostatic pneumonia and senility and with her fracture unhealed. There were no signs of malignant deposits, and the area where the ulcer had been remained soundly healed to the end.

Mrs. E. H—, a widow of 67 years, was referred to us for treatment to secondary neoplastic nodes in the right inguinal region. She had been treated by mid-thigh amputation, at another hospital, for a 30-year-old decubitus ulcer which, at the time of amputation, extended from the dorsum of the right foot to one-third of the distance up the tibial shaft and involved the bone. There had been no recent change in the ulcer prior to amputation but it had steadily increased in size during about 7 years before admission to hospital. Amputation had been advised for pain, and because of the large size and failure to heal with support and dressings.

On examination her right leg had been removed through the thigh. The skin flaps were nearly healed. Hard mobile nodes were present in both groins and in the right axilla. The left leg showed marked varicose veins and the scar of a healed ulcer above the left internal malleolus.

Her heart was fibrillating (she had been receiving treatment for this for about 6 years), but there were no signs of cardiac failure.

A report on sections taken from the ulcer after amputation showed a well-differentiated keratinising squamous-celled carcinoma.

A biopsy excision of nodes palpable in the axilla was performed. No malignant

---

EXPLANATION OF PLATES.

Fig. 1.—Photograph of the ulcer on Mrs. E. L—'s L. leg on admission. Biopsy was performed at the upper edge, which felt harder than surrounding parts.

Fig. 2.—Photograph of the ulcer on Mr. E. B—'s leg on admission.

Fig. 3.—Leg of Mr. E. B— after completion of radiotherapy.

Fig. 4.—Photograph of ulcer on Mrs. N. W—'s leg.

Fig. 5.—Photographs of ulcer on Mr. H. D—'s leg showing fungating cauliflower tumour.

Fig. 6.—Microphotograph of the biopsy of the tumour on Mr. H. D—'s leg. × 120.

Fig. 7.—Photograph of ulcer of Mr. J. R—. The tumorous growth can be plainly seen.

Fig. 8.—Radiograph of leg of Mr. J R—, prior to treatment, showing grows calcification in the body of the tumour with no apparent connection with the underlying bone, which shows only periostitis.

Fig. 9.—Microphotograph of original biopsy of ulcer on Mr. J. R—'s leg. The squamous epithelium of skin at the ulcer's edge may be seen. × 115.

Fig. 10.—High-power microphotograph of an area of the lymphatic secondary. × 120.

1

4

5

6

Black.

2

3

Black.

7

8

9

10

deposit could be found in these nodes. Biopsy of the nodes palpable in the right groin revealed a tumour similar in histology to the primary.

No further metastases were revealed by X ray of the spine, pelvis or lungs. Blood count was normal. A blood Wassermann reaction was not done.

Because of her general, and in particular, her cardiac condition extensive ablation of both groups of inguinal nodes was considered inadvisable. She was therefore submitted to deep X ray therapy (220 kv.) and a total skin dose of 4500 r given to each groin.

The groins, after showing moist desquamation, cleared quickly and the patient remains alive and well to date. This represents a survival time of 6 years.

Mr. H. D—, aged 58 years, was referred for treatment of an ulcer of the right leg. He had suffered from an ulcer at this site for 20 years, during which time it had never healed completely. Seven weeks before admission a swelling had developed in the upper part of the ulcer and had grown rapidly. This tumour had bled once before his admission (Fig. 5).

On examination there was a large ulcer of the right lower leg ; in the upper part of this ulcer there was a large fungating mass covered with a dark eschar. This mass was soft, friable and necrotic, about 2·5 in. in diameter. The tumour bled freely when touched. The right inguinal nodes were enlarged, discrete, not adherent to surrounding tissues and not tender. The left nodes were also enlarged and mobile.

His general condition was poor, by reason of hypotension and chronic nephritis. His blood urea was 208 mg., W.B.C. 18,000 per cmm., 89 per cent neutrophils and Wasserman reaction negative.

X ray of the right lower leg showed gross sclerosis of the tibial shaft, and an area of necrosis of the shaft subjacent to a large soft tissue swelling. No other radiological evidence of malignancy was found.

Biopsy of the tumour showed a general structure like granulation tissue, with well-formed capillaries and abundant reticulin. Cytologically it was definitely sarcomatous, with large, pleomorphic, usually spindle-shaped cells with large irregular often multiple nuclei and numerous mitoses. It seemed to be an example of fibrosarcoma (Fig. 6).

Although the size and histology of the growth and the necrosis of bone underlying made primary mid-thigh amputation the treatment of choice, his general condition was so bad that it was decided to attempt more by radiotherapy. Lateral and medial fields were used and skin doses of 3300 and 3000 r respectively given using 220 kv. However, only slight response to this therapy could be found on serial biopsy, the tumour cells and stroma remaining as in the first biopsy, but mitoses became less frequent and some collagen was deposited.

His condition deteriorated and he became anuric. His blood urea, reduced by diet and conservative measures, again rose above 200 mg. per cent. Ureteric catheterization and wash-outs produced no urine. Intra-venous isotonic sodium sulphate and bladder irrigation produced no change, and he died of uraemia one month after admission. Post-mortem revealed contracted fibrotic kidneys, an enlarged and flabby heart, pulmonary congestion with areas of bronchopneumonia, but no evidence of sarcomatous metastases could be detected.

Mr. J. R—, aged 76 years, had suffered from bilateral varicose veins with extensive chronic eczema of both legs and occasional ulceration of the left leg for over 30 years. Eighteen months prior to his admission a small lump appeared just above the right internal malleolus and this tumour grew and ulcerated. He presented with a florid fungating growth as shown in Fig. 7. He had never before noticed frank ulceration at this site.

On examination his general condition was excellent. Locally there was a large fungating ulcer over the right internal malleolus with heaped-up rolled edges and a deep sloughing crater. The whole mass appeared fixed deeply. There was one large, very hard node in the right groin. Extensive varicosities were present in both legs both in the long and short saphenous systems, with much scaling from eczema, and the scars of old ulcers were present on the left leg.

X ray of the right ankle revealed extensive calcification occurring in a soft tissue mass situated over the right internal malleolus, but this calcification did not appear to originate from the underlying bone, which showed only slight reactive periostitis (Fig. 8). Clinically there was no evidence if Paget's disease of bone, and no radiological evidence of this disease could be found in the skull spine or pelvis. The other long bones appeared normal also.

Biopsy was performed and the report read as follows : " Most of this is ulcerated and infected granulation tissue but one corner contains neoplastic tissue. It consists of solid rounded acellular blocks of tissue, staining pale pink with eosin, but a good red with van Gieson, between which columns run small groups of dark-staining polygonal cells, somewhat epithelial in appearance. Except that it is a tumour no certain diagnosis seems possible : the balance of opinion favours osteogenic sarcoma but a strong minority favours carcinoma. Further biopsy would be of assistance " (Fig. 9).

Several further biopsies were taken but no certain diagnosis could be given. Because of the patient's age and at his request cure was attempted by radiotherapy, but it soon became apparent that no regression was occurring and biopsy excision of the node in the groin was considered and performed. This node contained a nodule consisting entirely of masses of woven bone, in the interstices of which were dark-staining polygonal epithelial type cells very similar in appearance to the cells of the first biopsy (Fig. 10).

It was concluded that this tumour represented a most unusual and atypical form of osteogenic sarcoma, and the patient was advised to have a mid-thigh amputation and excision of the inguinal nodes. Because he lived alone and also owned a small personal business he refused to lose his leg above the knee, and we were constrained therefore to perform a below-knee amputation and block dissection of inguinal nodes. This dissection was performed with great thoroughness and a block of areolar tissue and nodes extending up to the bifurcation of the right common iliac artery was removed. Careful sectioning of this mass revealed no other metastasis.

Healing of the amputation flaps by first intention was rapid but the groin wound broke down. He refused skin grafting as he desired to get back to his business and discharged himself. The wound healed by granulation. He remained well for one year, when he was readmitted for treatment of a mass in the posterior flap of the stump. This mass was excised locally, a wide wedge of tissue being removed down to the bone. This local operation was again performed at the patient's insistence. Section of the tumour contained in this block of tissue revealed the identical histological picture presented by the original tumour and the nodule found in the inguinal lymph node. Healing was by first intention. He continued well until March, 1951, when he was admitted to another hospital. He was here discovered to have " cannon-ball " secondaries present in both lungs and he died July, 1951, about 4 years after the first operation, at the age of 82 years.

### COMMENT.

There has always been much discussion on the part played by chronic irritation in the aetiology of malignant disease. Whilst there can be no doubt of the significance of such irritants as tar and the carcinogenic hydrocarbons contained in it, of thorium and its bye-products, of radio-active earths, and of many other chemical products or physical energies in the production of malignant change, the position regarding inflammatory irritation is not so clear. Epithelioma of the lower limb is very uncommon in comparison with epithelioma elsewhere. This rarity must be associated with the fact that the legs are normally covered during adult life and thus protected from actinic radiation. At the same time, it is interesting to note that almost all the recorded cases of lower leg epithelioma have occurred in tissues subjected to years of irritation by chronic inflammatory processes. It would therefore appear that chronic inflammation may be of significance in the causation of cancer, but that the prodromal period is inordinately long.

With special reference to varicose ulcer or eczema it is impossible to disregard the views of Tenopyr and Silverman (1932), who maintain that an incidence of 0·4 per cent of malignant degeneration in such an extremely common condition as chronic varicose veins must indicate that these malignancies occurred by chance and not because of any precancerous propensities in the varicose condition.

We were struck by the observation of Gotheil (1912), which was supported by Knox (1925), that the dermatitis consequent upon varicose veins seems to be more commonly followed by malignant change than frank ulceration. In 2 of our cases the tumours occurred in non-ulcerated skin : that of Mrs. M. W—, in the scar of an old ulcer, and that of Mr. J. R— in deeply pigmented eczematous skin on a leg which had never previously suffered ulceration.

It was not possible accurately to assess the part played by local applications aetiologically—i.e., tar preparations, red lotion and other topical applications designed to increase granulation—because no patient could remember, from the infinite variety of applications tried over the years, what was contained in any of them. No case gave evidence of recent application of known carcinogenic substances.

It seems to us that varicose conditions of the legs, even if present for very many years, should not be considered as in any way pre-malignant. On the other hand progressive growth of the ulcer, the occurrence of haemorrhage, heaping-up or hardening of the edges or a hard node in the groin should be considered as pointers to a malignant change. There should be no hesitation in performing biopsy. There growths do not appear to present the characteristics of epitheliomata elsewhere, being slowly growing, slow to metastasise, and slow to kill, so that early treatment affords an excellent prognosis ; indeed it seems likely that the slow progression of these tumours often remains unremarked and the patient dies with an unrecognised malignant ulcer.

It was our belief, prior to personal experience with this type of case, that the treatment of choice was radiotherapy-controlled by serial biopsy. This form of treatment had been advocated in the literature (Rubenfeld, 1934), and it was in line with the treatment proposed for epitheliomata on other sites. Furthermore the age of the patient made the fitting of an artificial limb and education in the use of such a limb difficult and time-consuming procedures. However our experience with these cases has rather altered our view. In no less than 3 out of the 4 cases amputation had to be advised and, but for the death of one patient, would have been performed. Operation was forced upon us by the fact that, although the malignant disease had been eradicated by radiotherapy, the patients were still made utterly miserable by a large weeping, painful and foul-smelling ulcer showing no signs of healing at all. In 2 cases this condition prevented ambulation and the patient was bedridden. In these circumstances we would, in future, have no hesitation in recommending amputation, below-knee if it is possible to fashion flaps of healthy skin, otherwise mid-thigh, as immediate treatment. This will prevent loss of time, decrease the period of pain for the patient, and allow the patient to acclimatize himself to the loss of his leg without the disappointment of failed conservative treatment. Radiotherapy should be held in reserve for those patients whose general condition does not permit of operation, and these must be very few now.

The treatment of the inguinal nodes depends upon the age and general condition of the patient. Extensive block dissection is a formidable undertaking in the very old, and in these cases irradiation should be advised. Where the patient will bear the operation this represents the safest method of eradicating growth.

Sarcoma, largely owing to its extreme rarity is difficult to recognise both clinically and histologically. It appears to occur more towards the centre of an ulcer than at the edge, to produce a florid fungating cauliflower growth and to bleed readily. The most common histological type is a fibrosarcoma. These tumours do not react to irradiation and are best treated by amputation. As with the squamous growths, sarcomata in varicose ulcers appear less ready to metastasise than similar tumours elsewhere or in younger patients. This may be due to the fibrosis and scarring present after years of ulceration. The lymph-node metastasis report in the case of Mr. J. R— must be regarded as a pathological curiosity.

In conclusion we consider that this rare condition, if recognised and confirmed by biopsy at an early stage and actively and adequately treated, should have but little deleterious effect on the life expectation of the patient.

### SUMMARY.

Six cases of malignant degeneration occurring in varicose ulcer or eczema are reported.

The literature is reviewed, 69 cases of epitheliomata and 3 cases of sarcomata being collected. Four epitheliomata and 2 sarcomata are added. Aetiology and treatment are discussed.

We wish to thank Professor Ian Aird, Miss C. Wood and Mr. M. R. Ewing of the Postgraduate Medical School of London for permission to make use of material pertaining to their patients. We would also wish to thank Professor J. H. Dible and Dr. C. V. Harrison of the Dept. of Pathology for the histological specimens and their reports and Mr. E. V. Willmott, F.R.P.S., for his photographs.

### REFERENCES.

D'Assis, A.—(1926) *Ann. Surg.*, **83**, 663.
Donati, D.—(1935) *Rif. med.*, **51**, 865.
Glasser, J.—(1939) *Amer. J. Surg.*, **43**, 776.
Gothiel, W. S.—(1912) *J. Amer. med. Ass.*, **59**, 14.
Knox, L. C.—(1925) *Ibid.*, **85**, 1046.
Lang, C.—(1932) *Münch. med. Wschr.*, **79**, 1803.
Mondor, C.—(1938) *Soc. anat. Paris*, **314**, 15
Prewitt, T. F.—(1884) *J. Amer. surg. Ass.*, **2**, 491.
Rubenfeld, S.—(1934) *Amer. J. Surg.*, **26**, 372.
Tenopyr, J., and Silverman, I.—(1932) *Ann. Surg.*, **95**, 754.
Volkmann, R. von.—(1886–1890) *Samml. klin. Vortr. Chir.*, No. 335, p. 235.